# Objectification of Skin Glow: In Vivo Evaluation of 300 Women in Relation to Age

**DOI:** 10.1111/jocd.70373

**Published:** 2025-08-23

**Authors:** Alena Roessle, Martina Kerscher

**Affiliations:** ^1^ Cosmetic Science, Institute of Biochemistry and Molecular Biology University of Hamburg Hamburg Germany

**Keywords:** biophysical measurements, skin barrier, skin glow, skin physiology/structure, skin quality, statistics

## Abstract

**Objective:**

The influence of skin quality (SQ) on perceived attractiveness and skin health has been studied extensively. An international consensus has identified four “emergent perceptual categories” (EPCs) for SQ, which are linked to specific parameters. However, there is currently a lack of data on how these parameters correspond objectively with age in facial and nonfacial skin. This study aims to investigate the impact of age on the EPC skin glow and to establish objective reference ranges for five distinct age groups.

**Methods:**

A cohort of 300 Caucasian women, aged 20–69 years, was divided into five age groups. Skin glow was evaluated by measuring melanin (MI), erythema index (EI), and gloss diffuse scattering correction (DSC) value. The parameters were assessed with a Mexameter and a Skin‐Glossymeter (Courage+Khazaka electronic GmbH, Cologne, Germany) on five areas: the forehead, cheek, neck, décolleté, and the back of the hand.

**Results:**

A moderate positive correlation was observed between age and EI for the neck. Low correlations were identified for the neck (MI), décolleté (EI, gloss DSC), and hand (MI, EI, and gloss DSC). No correlations were found between age and the EI, MI, and gloss DSC parameters in the facial areas, nor for gloss DSC in the neck and MI in the décolleté.

**Conclusion:**

Age‐related changes in nonfacial areas, particularly the neck, highlight its potential as a reference site for skin assessment in Caucasians. Reference ranges could guide treatments for detractors like erythema and pigmentation. Future studies should consider external influences to refine intervention strategies.

## 1 Introduction

Skin quality (SQ) is a critical factor influencing individual well‐being and quality of life [1], as well as external perceptions of health and attractiveness [2]. This importance has driven a growing interest in various rejuvenation procedures and cosmeceuticals. The increase in search queries for cosmetic procedures in the United States, as shown by Google Trends tool data from 2018 to 2023, reflects this trend [3]. While the face remains the dominant focus of cosmetic care, nonfacial areas such as the neck, décolleté, and hands often receive less targeted intervention despite being commonly exposed and visible. These regions can therefore serve as alternative anatomical reference sites for detecting age‐related changes in SQ, particularly skin glow, with minimal interference from aesthetic treatments. To evaluate the effectiveness of minimally invasive procedures and topical products on the skin, biophysical measurements are commonly employed [4, 5]. However, the methods selected to evaluate SQ have varied across studies due to the lack of a comprehensive definition of SQ.

To address this gap, an international consensus to establish a standardized definition of SQ was reached. Goldie et al. define good quality of skin as appearing healthy, youthful, and free from damage. The consensus identified four emergent perceptual categories (EPCs). These are skin firmness, skin surface evenness, skin tone evenness, and skin glow. Each EPC is associated with specific parameters, except for skin glow, for which several synonyms have been described such as radiance, luminosity, brightness, vibrance, and complexion [6]. Around the same time, Humphrey et al. also emphasized the importance of standardizing a definition of SQ. The authors' proposed structure grouped the attributes of SQ into three categories: visual, mechanical, and topographical. Skin glow, defined by Humphrey et al. [7] as the skin's ability to reflect light, is highlighted as a critical attribute within the visual category.

Goodman et al. found that skin glow is determined by evaluating several detracting factors across the different layers of the skin that disrupt light reflectance. One category of these factors relates to skin color and includes erythema/redness, telangiectasia, pigmentation (such as lesional pigmentation, melasma, postinflammatory hyperpigmentation, and poikiloderma), and hypopigmentation. While aging introduces additional challenges to SQ, key characteristics of skin glow, such as an even skin tone, are influenced by various factors, some of which are independent of age [8]. Based on the methodologies proposed in the studies by Goldie et al. and Humphrey et al. [6, 7], skin glow can be quantitatively measured using a Glossymeter and Mexameter.

Skin glow is considered one of the most crucial elements influencing initial perceived attractiveness [8]. Despite its clinical relevance, it remains the least objectively defined and quantified of the four EPCs. To our knowledge, no large‐scale study has systematically examined skin glow across multiple anatomical sites in relation to age using validated, quantitative biophysical methods. The aim of the study is to objectively measure the EPC of skin glow with respect to age using the recommended biophysical measurement methods [6, 7]. We hypothesized that skin glow parameters would demonstrate age‐related changes across regions, more prominent in exposed regions such as the face. The study also seeks to define objective reference ranges that can support future clinical assessment and intervention planning.

## 2 Materials and Methods

All ethical principles and scientific regulations were followed in the conduct of this clinical trial. The research received approval from the independent ethics committee. A total of 300 Caucasian participants, aged 20–69 years, were enrolled in this study subsequent to obtaining written informed consent. Eligibility criteria are the same as detailed in Roessle and Kerscher [9] and provided in Table 1. Participants were stratified into five age cohorts with nine‐year intervals, each cohort comprising 60 female subjects. Demographic data distribution is shown in Table 2.

**TABLE 1:** Eligibility criteria as Roessle and Kerscher [9].

Eligibility criteria	
Inclusion criteria	Caucasians with Fitzpatrick skin type (FST) I–IV
	Age requirements 20–49 years: Premenopausal In the follicular phase of the menstrual cycle No recent changes in hormonal contraception (within the last 3 months) 50+ years: Postmenopausal No hormone replacement therapy for at least 1 year
	Consistent skin care routine for a minimum of 3 months
	Unchanged skin care routine for at least 3 months
	Healthy skin and overall stable health condition
Exclusion criteria	Body mass index under 18.5 or 30 and above
	Factors affecting the measurement area (e.g., scars, tattoos)
	History or current signs of tanorexia or severe sunburn
	Skin aging beyond typical expectations for the participant's age
	Pregnancy or breastfeeding
	High nicotine intake (≥ 20 cigarettes per week for over 2 years)
	Substance abuse, including excessive alcohol or drugs
	Any condition or treatment that could alter skin appearance: Phototherapy or chemotherapy within the past 6 months Use of prescription oral or topical antiaging/skin improvement products in the last 3 months Immunosuppressive or immunomodulatory medications, including systemic corticosteroids Cosmetic or medical skin treatments in the previous 3 months Any (minimally) invasive procedure at the measurement sites
	Smoking or caffeine consumption before or during the visit
	Application of topical products within 12 h prior to the visit
	Water contact on the measurement sites within 6 h before the visit
	Exposure to extreme temperatures (e.g., cryotherapy, sauna)
	Exercise or alcohol consumption within 24 h prior to the visit
	Participation in another clinical study within the past 30 days

**TABLE 2:** Demographic data.

Methods	Age groups	Age ranges in years	Mean age ± SD	Sample sizes	Skin types
Mexametry	AG I	20–29	25.0 ± 2.8	59	I: 10; II: 34; III: 11; IV: 4
	AG II	30–39	32.4 ± 2.3	52	I: 13; II: 26; III: 11; IV: 2
	AG III	40–49	44.2 ± 2.8	59	I: 12; II: 26; III: 15; IV: 6
	AG IV	50–59	55.1 ± 2.5	52	I: 9; II: 22; III: 17; IV: 4
	AG V	60–69	63.2 ± 2.8	53	I: 17; II: 22; III: 14
Glossymetry	AG I	20–29	25.1 ± 2.8	59	I: 10; II: 34; III: 11; IV: 4
	AG II	30–39	32.5 ± 2.4	60	I: 14; II: 33; III: 11; IV: 2
	AG III	40–49	44.3 ± 2.8	60	I: 13; II: 26; III: 15; IV: 6
	AG IV	50–59	54.9 ± 2.6	60	I: 10; II: 26; III: 20; IV: 4
	AG V	60–69	63.4 ± 2.7	59	I: 18; II: 25; III: 16

**FIGURE 1:**
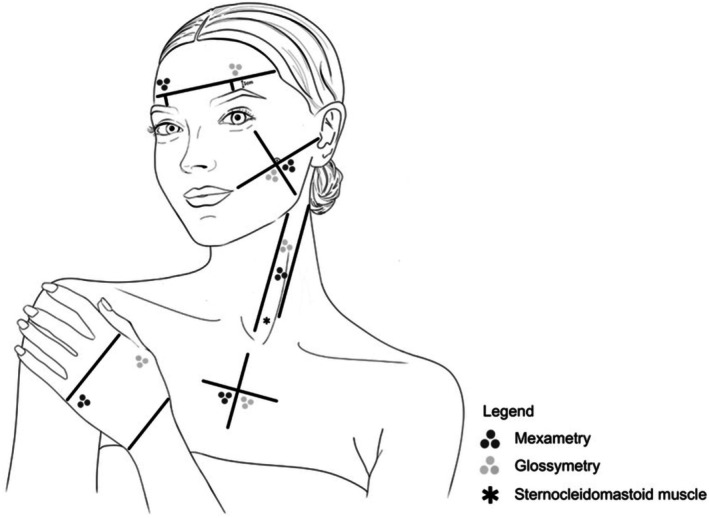
Skin areas for biophysical measurements adapted from Roessle and Kerscher [9].

Biophysical measurements to assess skin glow were performed during a single visit at the University of Hamburg between September 2022 and March 2024. Data were collected from the same five skin regions specified in the study by Roessle and Kerscher [9]: forehead, cheek, neck, décolleté, and dorsum of the hand (Figure 1). The left or right side of each area were chosen at random. Measurements were taken in controlled laboratory conditions to provide standardization (temperature 20.35°C ± 1.52°C, relative humidity 51.85% ± 6%), following a 30‐min acclimatization. Three measurements per skin area were taken by the same investigator with the subject in a supine position.

The Mexameter MX 18 (Courage+Khazaka electronic GmbH, Cologne, Germany) determines the melanin index (MI) and erythema index (EI) of the skin, as described in the literature [10, 11, 12]. The higher the respective values, the higher the melanin content in the skin or the more intense the erythema.

The Skin‐Glossymeter GL 200 (Courage+Khazaka electronic GmbH, Cologne, Germany) measures skin gloss based on the light that is reflected from the skin surface while applying a diffuse scattering correction (DSC) to minimize the influence of skin color [13].

The statistical analysis, as detailed in Roessle and Kerscher [9], used IBM SPSS v26 (IBM Corp., Armonk, NY, USA). Due to technical issues during data collection, only 275 Mexameter readings and 298 Glossymeter readings were obtained, resulting in slightly varying sample sizes across groups, as shown in Table 2. The missing data were assumed to be missing completely at random, and the analysis was conducted with the available datasets.

**FIGURE 2:**
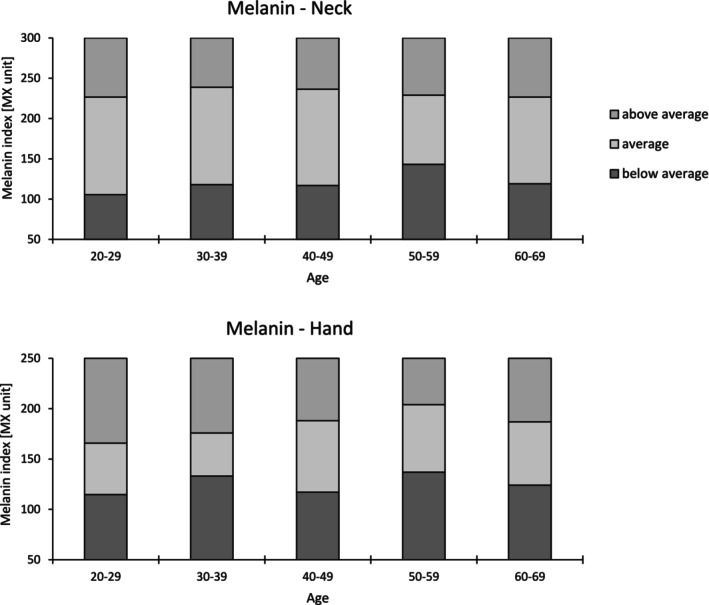
Reference values of melanin index for the neck and hand.

**FIGURE 3:**
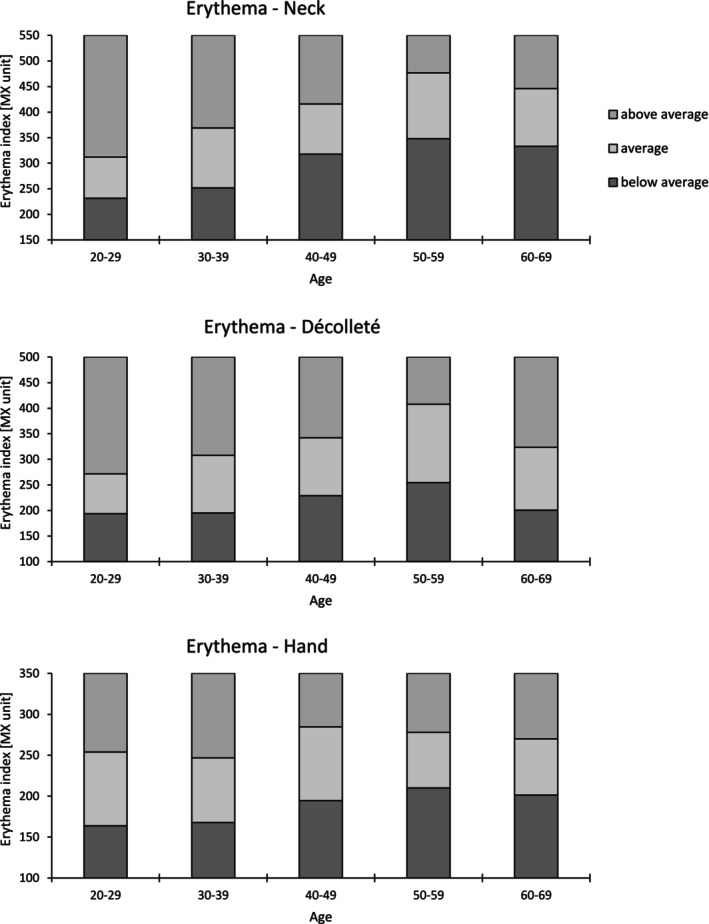
Reference values of erythema index for the neck, décolleté, and hand.

**FIGURE 4:**
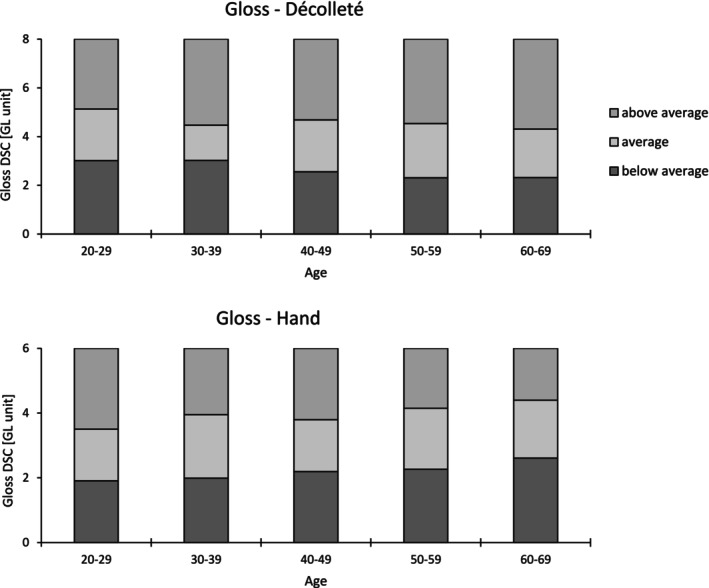
Reference ranges of gloss DSC for the décolleté and hand.

For each skin area, the average of the three recorded measurements was analyzed. The correlation between age and each parameter was examined using the Bravais–Pearson method, with Cohen's effect sizes categorized as small (*r* ≥ 0.1), moderate (*r* ≥ 0.3), and large (*r* ≥ 0.5). A significance threshold of 5% was applied. To objectify age‐related parameters regarding skin glow, reference ranges were established for parameters showing a significant Pearson correlation. Reference ranges, determined by the 25th (first quartile) and 75th (third quartile) percentiles was visualized graphically (Figures 2, 3, 4). All statistical test assumptions were satisfied. Mild and extreme outliers were reviewed in the raw data and retained as valid deviations.

## 3 Results

Table 3 shows the correlation coefficients of each parameter with age at each site. The descriptive data are provided in Table S1. As in Roessle and Kerscher [9], the interquartile range was used to set reference ranges for parameters with age‐related correlations, categorized as below average, average, or above average and displayed in Figures 2, 3, 4.

**TABLE 3:** Overview of the correlation coefficients between age and each parameter.

Pearson correlation		Facial skin		Nonfacial skin		
		Forehead	Cheek	Neck	Décolleté	Hand
Age	Melanin index	−0.098	−0.011	0.153**	−0.001	0.206**
	Erythema index	0.079	−0.037	0.457**	0.169**	0.220**
	Gloss DSC	0.079	0.078	−0.052	−0.207**	0.166**

*Note:* Significant is marked with **p < 0.01.

### 3.1 Forehead

There were no correlations with age observed for MI (*r* = −0.098, *p* > 0.05), EI (*r* = 0.079, *p* > 0.05), or gloss DSC value (*r* = 0.079, *p* > 0.05).

### 3.2 Cheek

Similarly, no correlations with age were found for MI (*r* = −0.011, *p* > 0.05), EI (*r* = −0.037, *p* > 0.05), or the gloss DSC value (*r* = 0.078, *p* > 0.05).

### 3.3 Neck

A moderate but significant positive correlation with age was found for EI (*r* = 0.457, *p* < 0.01). A low but significant correlation was identified for MI (*r* = 0.153, *p* < 0.01). No correlation was observed for the gloss DSC value (*r* = −0.052, *p* > 0.05).

### 3.4 Décolleté

Low but significant correlations with age were found for EI (*r* = 0.169, *p* < 0.01) and the gloss DSC value (*r* = −0.207, *p* < 0.01). MI (*r* = −0.001, *p* > 0.05) showed no correlation with age.

### 3.5 Hand

Low but significant correlations with age were observed for all parameters: MI (*r* = 0.206, *p* < 0.01), EI (*r* = 0.220, *p* < 0.01), and gloss DSC value (*r* = 0.166; *p* < 0.01).

## 4 Discussion

Skin glow is one of the four EPCs defining SQ [6], yet its quantification in relation to age remains challenging. While objective data exist for the EPC skin firmness [9], skin glow is less amenable to standardization due to its complex combination of factors and the multifactorial influences on its perception. Goodman et al. [8] highlighted 15 detractors of skin glow at various skin layers, including erythema in the papillary dermis and pigmentation/melasma at the dermal/epidermal junction. These two detractors align with the measurements recommended by Goldie et al. [6] which were examined in this study as EI, MI, and gloss DSC. Only these parameters were selected based on their biophysical measurement relevance for skin glow, as outlined by Goldie et al. [6]. However, this focus inherently limits the inclusion of additional parameters identified as skin glow detractors by Goodman et al. [8].

Interestingly, in the facial area (forehead, cheek), the correlation coefficients between age and all three parameters (EI, MI, and gloss DSC) were close to zero, with no significance. Since the face is frequently the primary focus of beauty and character [14], this finding may reflect increased attention to facial appearance. It is reasonable to assume that even small changes in SQ in the face may be noticed immediately and treated, leading to improved skin glow regardless of age. This increased focus on facial appearance is evidenced by the ever‐increasing variety of facial treatments and products on the market that target SQ issues [15].

Another consideration is the possible influence of measurement site placement. The cheek measurements in this study were taken laterally, which may not fully capture the centrofacial zone where erythema and pigment changes typically manifest with age. Similarly, facial areas exposed to makeup or skincare residue, even with washout periods, may result in less accurate readings for surface‐based devices like the Glossymeter. These technical and anatomical factors, combined with behavioral patterns in aesthetic care, likely contribute to the absence of aging‐related trends in facial skin glow parameters in this cohort.

In nonfacial areas, significant correlations were found between age and MI, EI, and gloss DSC. Significant correlations between EI and age were observed for the neck, décolleté, and hand, with the most notable correlation on the neck (*r* = 0.457, *p* < 0.01). Earlier research has identified variations in erythema based on age groups, anatomical regions, and gender. However, the comparability of these studies is limited. Data from Nedelec et al. indicated that EI is generally higher in all subject groups compared to our findings, possibly due to differences in the selection of ethnicities. The forehead and cheek exhibited the highest values, aligning with findings from Kleesz et al., who analyzed biophysical skin parameters across 16 skin areas [16, 17]. In our study, the highest values were found on the neck (AG III: 362.16 ± 79.29 EI unit; AG IV: 407.80 ± 90.54 EI unit; AG: V 382.99 ± 83.15 EI unit).

As the development of erythema is multifactorial [18], it is difficult to define regional and age‐related reference values. A Belgian study of 137 subjects concluded that the regional variability of EI is too varied to be predictable [19]. As our study measured only Caucasians with a relatively homogeneous group of Fitzpatrick skin types (FSTs), our study is the first to show a significant moderate correlation between EI and age at the neck for this population.

MI showed only low correlations with age for the neck and hand, with the highest coefficient observed at the hand (*r* = 0.206, *p* < 0.01). This aligns with the hands' unique exposure to environmental and mechanical stressors, such as UV radiation and frequent washing. In addition to intrinsic factors such as genetics and pathologies, extrinsic factors like traumatic injury, chemical irritation, and UV light often play a decisive role in SQ [20]. UVA1 wavelengths in particular contribute to the appearance of clinical aging changes, such as hyperpigmentation [21]. The age‐related correlations of EI and MI on the neck and on the décolleté may have been subdued, likely due to protective factors such as Western clothing norms and the limited number of sunny days the Germany‐based cohort is exposed to.

Although the correlation coefficients were low, the décolleté showed an age‐related decrease in the gloss DSC value, while the hand showed an increase. Since hands were not washed and topicals were not applied prior to the measurements (for exact details see Table 1), a possible influence of shimmering residues of cosmetics or other particles cannot be completely excluded.

This examination was conducted on a relatively uniform population of Caucasian females with healthy skin, which may limit the applicability of the findings to other ethnic groups and skin types. Seasonal variability, despite efforts to control for it, may have subtly influenced results, as measurements were predominantly taken during winter months. Additionally, the diurnal rhythm of skin barrier parameters, as reported by Ostermeier et al. [22], could have influenced EI measurements, emphasizing the need for standardization in timing for future studies.

Furthermore, the study encountered missing data: 275 of 300 Mexameter readings and 298 of 300 Glossymeter readings were available for analysis. While these gaps were relatively small and randomly distributed, their potential impact on the robustness of the correlation analyses, particularly in regions such as the hand and neck, where correlation coefficients were low to moderate, must be acknowledged. Reduced sample size in these subsets may have slightly limited statistical power and sensitivity, potentially obscuring associations or inflating variability. No patterns in the missing data were observed that would suggest systematic bias. Additionally, while the study applied strict inclusion and exclusion criteria to reduce variability, we did not account for lifestyle factors known to influence SQ, such as cumulative sun exposure, use of sun protection, smoking history, or detailed skincare routines. These external variables may contribute to interindividual differences in erythema, pigmentation, and gloss values. Future studies should incorporate these confounders into multivariate analyses to refine the interpretation of age‐related changes in skin glow.

To conclude, our study provides initial insights into the correlation between age and biophysical parameters of skin glow, as recommended by Goldie et al. [6] Objective measurements of skin glow remain a complex challenge due to the many factors that influence it. Understanding how these factors interact and change with age requires a comprehensive approach. However, establishing reference ranges divided into three classifications, as in Roessle and Kerscher [9], can provide valuable insights into SQ assessment and guide the selection of targeted treatments.

By targeting specific detractors of skin glow, these treatments can help mitigate negative influences and enhance overall SQ. Injectables with a demonstrated ability to improve skin glow, such as hyaluronic acid (HA), cohesive polydensified matrix HA with glycerol (CPM‐HA20G), and diluted calcium hydroxylapatite [5, 6, 23], as well as hybrid fillers, may be suitable candidates.

Measurements for the EPC “Skin Glo”, such as melanin and erythema indices, are also used in the assessment of other EPCs. This overlap illustrates the inherent challenge of differentiating skin glow as a distinct construct and reinforces the interconnectedness of the EPCs. As a result, treatments intended for one EPC often confer benefits across multiple domains. For example, transdermal injections of HA‐based gel fillers not only improve skin gloss but can also enhance elasticity, hydration, pore size, and fine lines [24]. Furthermore, intradermal injections of botulinum toxin have been associated with reductions of erythema, highlighting additional avenues to enhance overall SQ [25]. In this context, a recent study in Asian populations demonstrated that treatment with CPM‐HA20G resulted in significant, objective improvements across all four EPCs, sustained up to 24 weeks [26]. These findings suggest an evolving body of evidence that some injectables, traditionally seen as single‐modality treatments, may offer broader benefits across multiple dimensions of SQ, including parameters associated with skin glow.

In addition to injectables, noninvasive topical products containing vitamin C, niacinamide, and HA can be utilized to improve erythema and pigmentation—key detractors of skin glow [4, 6]. Such topical interventions may be especially relevant for patients seeking gradual or preventative improvements in skin glow, particularly in early or subclinical stages of age‐related change.

In the present study, the neck emerged as a key site for age‐related changes in erythema, supporting its utility as a potential reference region for assessing age‐related changes to skin glow in Caucasian populations. Given its clinical accessibility and lower likelihood of prior aesthetic intervention compared to the face, the neck may serve as a valuable site for evaluating treatment response or for tracking longitudinal changes in SQ.

Looking ahead, future studies should build on these findings by incorporating broader lifestyle and environmental variables, including cumulative sun exposure, skincare behaviors, and smoking history, into multivariate models. This will help refine our understanding of age‐related SQ dynamics and better account for interindividual variability. Expanding the population to include other ethnic groups and a wider range of Fitzpatrick skin types will also be critical for generalizing reference ranges and optimizing treatment strategies. Ultimately, the establishment of standardized, objective benchmarks for skin glow, alongside the recognition of its overlap with other EPCs, represents an important step toward more comprehensive, individualized SQ assessment and care.

## Author Contributions

The author, Alena Roessle, is responsible for the conception, design, data collection, data analysis, and writing of this manuscript. Prof. Kerscher provided guidance and advisory support throughout the study process.

## Disclosure

The lead author, Alena Roessle, and the corresponding author, Martina Kerscher, affirm that this manuscript is an honest, accurate, and transparent account of the study being reported; that no important aspects of the study have been omitted; and that any discrepancies from the study as planned have been explained.

## Ethics Statement

The authors confirm that the ethical policies of the journal, as outlined on the journal's author guidelines page, have been adhered to, and appropriate approval from the ethical review committee has been obtained. The Independent Ethics Committee (Ethikkommission der Ärztekammer Hamburg) in Germany approved the study (2022‐100840‐BO‐ff) on September 20, 2022. The study was conducted in accordance with the principles of the Declaration of Helsinki and the International Conference on Harmonization Guidelines for Good Clinical Practice. Measurements were not taken prior to the signing of the informed consent.

## Conflicts of Interest

The authors declare no conflicts of interest.

## Supporting information


**Table S1:** jocd70373‐sup‐0001‐Supinfo.docx.

## Data Availability

The data that support the findings of this study are available from the corresponding author upon reasonable request.

## References

[1] A. Hosthota , S. Bondade , and V. Basavaraja , “Impact of Acne Vulgaris on Quality of Life and Self‐Esteem,” Cutis 98, no. 2 (2016): 121–124. 27622255

[2] N. Samson , B. Fink , P. J. Matts , N. C. Dawes , and S. Weitz , “Visible Changes of Female Facial Skin Surface Topography in Relation to Age and Attractiveness Perception: Perception of Facial Skin Surface Topography,” Journal of Cosmetic Dermatology 9, no. 2 (2010): 79–88. 20618552 10.1111/j.1473-2165.2010.00489.x

[3] A. J. Oh and D. B. Rootman , “Recent Fluctuations in Public Searches for Cosmetic Procedures as Shown by Google Trends,” Ophthalmic Plastic and Reconstructive Surgery 40 (2023): 266–269, https://journals.lww.com/10.1097/IOP.0000000000002562. 37972973 10.1097/IOP.0000000000002562

[4] P. Rattanawiwatpong , R. Wanitphakdeedecha , A. Bumrungpert , and M. Maiprasert , “Anti‐Aging and Brightening Effects of a Topical Treatment Containing Vitamin C, Vitamin E, and Raspberry Leaf Cell Culture Extract: A Split‐Face, Randomized Controlled Trial,” Journal of Cosmetic Dermatology 19, no. 3 (2020): 671–676. 31975502 10.1111/jocd.13305 PMC7027822

[5] D. Hertz‐Kleptow , A. Hanschmann , M. Hofmann , T. Reuther , and M. Kerscher , “Facial Skin Revitalization With CPM‐HA20G: An Effective and Safe Early Intervention Treatment,” Clinical, Cosmetic and Investigational Dermatology 12 (2019): 563–572. 31496779 10.2147/CCID.S209256 PMC6698156

[6] K. Goldie , M. Kerscher , S. G. Fabi , et al., “Skin Quality – A Holistic 360° View: Consensus Results,” Clinical, Cosmetic and Investigational Dermatology 14 (2021): 643–654. 34163203 10.2147/CCID.S309374 PMC8214518

[7] S. Humphrey , S. Manson Brown , S. J. Cross , and R. Mehta , “Defining Skin Quality: Clinical Relevance, Terminology, and Assessment,” Dermatologic Surgery 47 (2021): 974–981, https://journals.lww.com/10.1097/DSS.0000000000003079. 34148998 10.1097/DSS.0000000000003079 PMC8231670

[8] G. J. Goodman , K. Armour , D. Ong , et al., “An Absence of Imperfections: A Proposed Framework for Defining, Assessing, and Achieving Skin Glow,” Journal of Cosmetic Dermatology 23, no. 1 (2024): 161–171. 37929650 10.1111/jocd.16063

[9] A. Roessle and M. Kerscher , “Objectification of Skin Firmness: In Vivo Evaluation of 300 Women in Relation to Age,” Journal of Cosmetic Dermatology 24, no. 1 (2025): e16773. 39780520 10.1111/jocd.16773 PMC11712028

[10] M. Baquié and B. Kasraee , “Discrimination Between Cutaneous Pigmentation and Erythema: Comparison of the Skin Colorimeters Dermacatch and Mexameter,” Skin Research and Technology 20, no. 2 (2014): 218–227. 24033842 10.1111/srt.12109

[11] G. E. Piérard , “EEMCO Guidance for the Assessment of Skin Colour,” Journal of the European Academy of Dermatology and Venereology 10, no. 1 (1998): 1–11. 9552751 10.1016/s0926-9959(97)00183-9

[12] Z. Isa , K. Shamsuddin , N. B. I. Bukhari , et al., “The Reliability of Fitzpatrick Skin Type Chart Comparing to Mexameter (Mx 18) in Measuring Skin Color Among First Trimester Pregnant Mothers in Petaling District, Malaysia,” Malaysian Journal of Public Health Medicine 16, no. 3 (2016): 59–65.

[13] Courage + Khazaka electronic GmbH , “Information and Instruction Manual for the Skin‐Glossymeter GL 200 Probe,” Cologne (2022).

[14] A. Synnott , “The Beauty Mystique,” Facial Plastic Surgery 22, no. 3 (2006): 163–174. 17048156 10.1055/s-2006-950173

[15] M. A. Nilforoushzadeh , M. A. Amirkhani , P. Zarrintaj , et al., “Skin Care and Rejuvenation by Cosmeceutical Facial Mask,” Journal of Cosmetic Dermatology 17, no. 5 (2018): 693–702. 30133135 10.1111/jocd.12730

[16] B. Nedelec , N. J. Forget , T. Hurtubise , et al., “Skin Characteristics: Normative Data for Elasticity, Erythema, Melanin, and Thickness at 16 Different Anatomical Locations,” Skin Research and Technology 22, no. 3 (2016): 263–275. 26333046 10.1111/srt.12256

[17] P. Kleesz , R. Darlenski , and J. W. Fluhr , “Full‐Body Skin Mapping for Six Biophysical Parameters: Baseline Values at 16 Anatomical Sites in 125 Human Subjects,” Skin Pharmacology and Physiology 25, no. 1 (2012): 25–33. 21912200 10.1159/000330721

[18] R. Abdlaty and Q. Fang , “Skin Erythema Assessment Techniques,” Clinics in Dermatology 39, no. 4 (2021): 591–604. 34809765 10.1016/j.clindermatol.2021.03.006

[19] J. F. Hermanns , L. Petit , T. Hermanns‐Lê , and G. E. Piérard , “Analytic Quantification of Phototype‐Related Regional Skin Complexion,” Skin Research and Technology 7, no. 3 (2001): 168–171. 11554703 10.1034/j.1600-0846.2001.70305.x

[20] E. Carmeli , H. Patish , and R. Coleman , “The Aging Hand,” Journals of Gerontology, Series A: Biological Sciences and Medical Sciences 58, no. 2 (2003): M146–M152. 10.1093/gerona/58.2.m146 12586852

[21] F. Bernerd , T. Passeron , I. Castiel , and C. Marionnet , “The Damaging Effects of Long UVA (UVA1) Rays: A Major Challenge to Preserve Skin Health and Integrity,” International Journal of Molecular Sciences 23, no. 15 (2022): 8243. 35897826 10.3390/ijms23158243 PMC9368482

[22] M. Ostermeier and M. Kerscher , “Der Diurnale Rhythmus der Haut: Mythos oder Realität?: Evaluation Mittels Biopyhsikalischer Messmethoden,” Aktuelle Dermatologie 44, no. 12 (2018): 539–546.

[23] Y. Y. Chao , J. W. Kim , J. Kim , H. Ko , and K. Goldie , “Hyperdilution of CaHA Fillers for the Improvement of Age and Hereditary Volume Deficits in East Asian Patients,” Clinical, Cosmetic and Investigational Dermatology 11 (2018): 357–363. 30038511 10.2147/CCID.S159752 PMC6053172

[24] J. H. Lee , J. Kim , Y. N. Lee , et al., “The Efficacy of Intradermal Hyaluronic Acid Filler as a Skin Quality Booster: A Prospective, Single‐Center, Single‐Arm Pilot Study,” Journal of Cosmetic Dermatology 23, no. 2 (2024): 409–416. 37705328 10.1111/jocd.15944

[25] V. Dicker , M. D. M. Serna Posada , E. Galeano Piedrahita , S. Vasquez Villegas , and C. Perez Madrid , “Intradermal Microdroplet Injection of Standard‐Diluted Incobotulinumtoxin A for the Treatment of Erythematotelangiectatic Rosacea: A Study From a Dermatology Center in Medellín, Colombia,” Cureus 17 (2025), https://www.cureus.com/articles/325846‐intradermal‐microdroplet‐injection‐of‐standard‐diluted‐incobotulinumtoxin‐a‐for‐the‐treatment‐of‐erythematotelangiectatic‐rosacea‐a‐study‐from‐a‐dermatology‐center‐in‐medelln‐colombia. 10.7759/cureus.78957 PMC11910203 40092010

[26] J. Park , C. Youn , C. Lee , et al., “Facial Skin Quality Improvement After Treatment With CPM – HA20G: Clinical Experience in Korea,” Journal of Cosmetic Dermatology 24, no. 1 (2025): e16795. 39844659 10.1111/jocd.16795 PMC11755000

