# Supplementary material for: Objectification of Skin Glow: In Vivo Evaluation of 300 Women in Relation to Age
Source: J Cosmet Dermatol. 2025 Aug 23;24(Suppl 4):e70373. doi: 10.1111/jocd.70373 (PMC12374564; doi:10.1111/jocd.70373)
Supplement: Supplementary file 1 — Table S1: jocd70373‐sup‐0001‐Supinfo.docx. [file JOCD-24-e70373-s001.docx]

Supplemental Table 1: Mean Value ± SD and Median with quartiles of the age groups per AREA.

| **Parameter** | **Site** | | | | |
| --- | --- | --- | --- | --- | --- |
|  | **Forehead** | **Cheek** | **Neck** | **Décolleté** | **Hand** |
| **Melanin index [MX unit]** | | | | | |
| AG I  n=59 | 150.92±45.57  Q_1_=121.00  Med=143.33  Q_3_=180.67 | 151.19±43.48  Q_1_=119.33  Med=145.00  Q_3_=172.67 | 146.67±51.75  Q_1_=105.67  Med=140.67  Q_3_=179.00 | 130.42±50.01  Q_1_=94.67  Med=123.67  Q_3_=160.67 | 147.8±42.81  Q_1_=114.67  Med=146.67  Q_3_=165.67 |
| AG II  n=52 | 156.03±43.44  Q_1_=138.17  Med=159.00  Q_3_=175.83 | 155.62±42.99  Q_1_=126.83  Med=152.84  Q_3_=176.25 | 152.28±46.93  Q_1_=118.09  Med=153.84  Q_3_=179.33 | 136.14±51.56  Q_1_=94.08  Med=142.17  Q_3_=169.17 | 142.34±44.78  Q_1_=114.50  Med=148.00  Q_3_=165.33 |
| AG III  n=59 | 154.81±52.51  Q_1_=118.67  Med=144.67  Q_3_=183.00 | 155.98±47.46  Q_1_=121.00  Med=147.00  Q_3_=173.33 | 158.88±59.02  Q_1_=117.00  Med=147.67  Q_3_=180.67 | 133.38±60.78  Q_1_=88.67  Med=115.67  Q_3_=159.67 | 155.34±57.44  Q_1_=117.00  Med=141.67  Q_3_=188.00 |
| AG IV  n=52 | 153,75±47.97  Q_1_=120.91  Med=151.67  Q_3_=179.67 | 163.17±41.15  Q_1_=130.75  Med=163.84  Q_3_=193.00 | 182.72±50.48  Q_1_=143.25  Med=182.34  Q_3_=214.25 | 148.28±54.74  Q_1_=111.17  Med=152.17  Q_3_=191.17 | 176.35±47.32  Q_1_=143.34  Med=175.83  Q_3_=204.75 |
| AG V  n=53 | 133.78±46.26  Q_1_=102.17  Med=135.00  Q_3_=168.84 | 143.43±44.23  Q_1_=110.00  Med=149.33  Q_3_=179.00 | 159.46±51.33  Q_1_=119.00  Med=159.33  Q_3_=192.50 | 123.42±47.30  Q_1_=85.00  Med=126.00  Q_3_=155.67 | 165.41±50.46  Q_1_=124.00  Med=160.67  Q_3_=187.00 |
| **Erythema index [MX unit]** | | | | | |
| AG I  n=59 | 291.85±57.77  Q_1_=250.00  Med=291.67  Q_3_=332.00 | 285.56±62.25  Q_1_=238.00  Med=284.67  Q_3_=335.00 | 280.97±71.83  Q_1_=231.67  Med=269.67  Q_3_=312.00 | 241.82±78.50  Q_1_=193.67  Med=227.33  Q_3_=271.67 | 211.56±60.03  Q_1_=163.67  Med=202.00  Q_3_=254.00 |
| AG II  n=52 | 293.08±62.75  Q_1_=252.67  Med=295.00  Q_3_=332.34 | 303.47±62.04  Q_1_=260.50  Med=294.84  Q_3_=332.75 | 317.05±77.00  Q_1_=251.92  Med=311.50  Q_3_=368.84 | 254.01±87.98  Q_1_=195.33  Med=249.50  Q_3_=307.92 | 212.85±52.16  Q_1_=167.92  Med=207.83  Q_3_=246.67 |
| AG III  n=59 | 319.08±62.98  Q_1_=277.33  Med=314.67  Q_3_=364.67 | 324.62±72.06  Q_1_=281.00  Med=312.33  Q_3_=379.67 | 362.16±79.29  Q_1_=318.00  Med=360.33  Q_3_=416.00 | 286.02±80.49  Q_1_=229.00  Med=299.00  Q_3_=342.00 | 235.91±58.09  Q_1_=194.67  Med=223.67  Q_3_=284,67 |
| AG IV  n=52 | 314.44±58.04  Q_1_=279.67  Med=318.34  Q_3_=350.5 | 311.07±59.30  Q_1_=274.67  Med=300.84  Q_3_=340.17 | 407.80±90.54  Q_1_=348.25  Med=411.67  Q_3_=476.84 | 326.44±102.92  Q_1_=254.75  Med=319.17  Q_3_=407.83 | 252.76±61.16  Q_1_=210.08  Med=246.67  Q_3_=278.00 |
| AG V  n=53 | 294.14±71.71  Q_1_=241.33  Med=282.33  Q_3_=338.83 | 271.32±68.39  Q_1_=232.00  Med=264.00  Q_3_=305.17 | 382.99±83.15  Q_1_=333.17  Med=382.00  Q_3_=446.00 | 257.80±79.98  Q_1_=200.83  Med=247.33  Q_3_=323.34 | 233.17±60.53  Q_1_=201.33  Med=222.33  Q_3_=270.00 |
| **Gloss DSC [GL unit]** | | | | | |
| AG I  n=59 | 5.65±2.28  Q_1_=3.90  Med=5.33  Q_3_=7.03 | 4.62±2.18  Q_1_=3.24  Med=4.36  Q_3_=5.93 | 1.84±0.78  Q_1_=1.21  Med=1.90  Q_3_=2.49 | 4.23±1.58  Q_1_=3.02  Med=4.02  Q_3_=5.13 | 2.82±1.20  Q_1_=1.91  Med=2.75  Q_3_=3.5 |
| AG II  n=60 | 5.92±2.10  Q_1_=4.56  Med=5.90  Q_3_=7.38 | 4.14±1.89  Q_1_=2.61  Med=4.07  Q_3_=5.22 | 1.65±0.86  Q_1_=1.00  Med=1.54  Q_3_=2.29 | 3.90±1.42  Q_1_=3.03  Med=3.87  Q_3_=4.47 | 3.05±1.28  Q_1_=1.99  Med=2.84  Q_3_=3.95 |
| AG III  n=60 | 5.68±1.89  Q_1_=4.29  Med=5.63  Q_3_=7.32 | 4.68±2.45  Q_1_=2.77  Med=4.32  Q_3_=6.01 | 1.63±0.97  Q_1_=1.03  Med=1.49  Q_3_=2.05 | 3.74±1.66  Q_1_=2.55  Med=3.64  Q_3_=4.69 | 2.93±1.14  Q_1_=2.19  Med=2.97  Q_3_=3.80 |
| AG IV  n=60 | 5.99±1.85  Q_1_=4.92  Med=5.98  Q_3_=6.99 | 4.89±1.99  Q_1_=3.43  Med=4.67  Q_3_=6.34 | 1.48±0.90  Q_1_=0.80  Med=1.25  Q_3_=2.16 | 3.59±1.40  Q_1_=2.31  Med=3.66  Q_3_=4.54 | 3.29±1.43  Q_1_=2.26  Med=3.18  Q_3_=4.15 |
| AG V  n=59 | 6.26±1.89  Q_1_=4.70  Med=6.23  Q_3_=7.87 | 4.86±2.00  Q_1_=4.05  Med=4.88  Q_3_=6.11 | 1.77±0.85  Q_1_=1.11  Med=1.65  Q_3_=2.41 | 3.25±1.34  Q_1_=2.32  Med=3.06  Q_3_=4.31 | 3.55±1.29  Q_1_=2.61  Med=3.45  Q_3_=4.40 |
